# The Coating Effect of Persian Gum (Zedo Gum) Containing *Lactobacillus sakei* on the Beef Quality Parameters During Storage at Refrigerator Temperature

**DOI:** 10.1002/fsn3.70024

**Published:** 2025-02-06

**Authors:** Mohammad Hasan Nemati, Shohreh Alian Samakkhah, Razieh Partovi, Abbas Isvand

**Affiliations:** ^1^ Department of Food Hygiene, Faculty of Veterinary Medicine Amol University of Special Modern Technologies (AUSMT) Amol Iran; ^2^ Department of Food Hygiene and Quality Control, Faculty of Veterinary Medicine Shahrekord University Shahrekord Iran

**Keywords:** beef, bioactive packaging, *Lactobacillus sakei*, Persian gum

## Abstract

Coatings with antibacterial properties, integrated with biological agents, offer a novel and promising strategy for preserving meat products. This study investigates the effect of Persian gum (PG) coating containing *Lactobacillus sakei* bacteria on beef quality during refrigerated storage. Beef loin pieces were divided into five groups (control, 1% PG, 2% PG, and 1% and 2% PG with *L. sakei* bacteria). The groups were evaluated for microbial, chemical, and sensory tests at specific periods (days 0, 2, 4, 6, and 8). The results of the microbial analysis (the mean LAB count) revealed that the quality of meat significantly (*p* < 0.05) improved in the presence of *L. sakei* coatings, ranging from 6.08 to 7.31 log_10_ CFU/g in different treatment groups at the end of the experiment. Additionally, coatings containing *L. sakei* significantly (*p* < 0.05) reduced the microbial counts of mesophilic, psychrophilic, and Enterobacteriaceae bacteria, resulting in an extended shelf life of at least 8 days. The chemical findings indicated that increases in pH values (ranging from 5.98 to 6.57), total volatile basic nitrogen (TVB‐N) levels (from 18.30 to 32.33 mg N/g), thiobarbituric acid reactive substances (TBARs) (from 2.16 to 4.12 mg MDA/kg), and protein carbonyl (PC) concentrations (from 1.33 to 2.05 nmol/mg protein) during storage at 4°C were ranked as follows: PG 2% + *L. sakei* < PG 1% + *L. sakei* < PG 2% < PG 1% < control. Additionally, overall acceptability, texture, odor, and color were significantly higher in the groups coated with *L. sakei* than in other groups. Based on the results, the groups covered with PG and *L. sakei* indicated that the quality and safety of beef increased and extended the shelf life of meat. In conclusion, PG solution containing *L. sakei* bacteria can be recommended as a new method for beef packaging.

## Graphical abstract

This study provides information about the use of a Persian gum solution containing *Lactobacillus sakei* bacteria for beef packaging. According to the results, the microbiological, chemical, and sensory analyses of the 2% Persian gum coating with *Lactobacillus sakei* bacteria revealed an increase in the shelf life of the meat by 2, 4, and 2 days, respectively, compared to the control group under refrigerated storage conditions. Based on the results, the 2% Persian gum solution containing *Lactobacillus sakei* bacteria can be recommended as a new method for beef packaging.

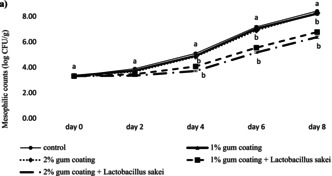

## 1 Introduction

The richness of beef in various vitamins such as B vitamins, minerals such as iron and zinc, and essential amino acids has led to its classification as one of the best and most complete food products. However, this protein‐nutritious nature, along with high moisture and pH, can become a weakness of this product and provide a very suitable environment for the growth and proliferation of pathogens. Naturally, with these conditions, even small microbial contamination can lower the shelf life of the product and threaten the consumer's health (Tayengwa et al. 2020).

Polysaccharides are commonly employed in the production of films and coatings due to their effective film‐forming abilities. Among the diverse range of carbohydrate polymers available, Persian gum (or Zedo gum) emerges as a highly promising candidate for the development of bio‐based antimicrobial coatings (Seyfi et al. 2019). PG, which is also known as Zedo gum, Angum gum, and Shirazi gum in different communities, exudes from the trunk of the mountain almond tree with the scientific name *Amygdalus scoparia*. The resulting gum is utilized in several industries in developed countries, including the food industry and pharmaceutical production, where it is employed for purposes such as alleviating toothache and reducing joint swelling. However, despite its potential, its use in traditional medicine in Iran remains limited. A significant portion of the gum, approximately 400 tons annually, is exported at a relatively low price (Khalesi et al. 2016). This gum, which is a non‐starch hydrocolloid with an acidic polysaccharide nature, naturally consists of two parts: water‐soluble (about 30%) and water‐insoluble (about 70%). The soluble part easily dissolves in cold water, but the insoluble part is only partially soluble in hot water. The structure of the soluble part is mainly composed of galactose sugars and a smaller amount of arabinose (in an approximate ratio of 1:2). This part can be used to prepare edible films and coatings (Dabestani et al. 2017). Several studies have been conducted to investigate the effect of gums, especially PG, on the shelf life of protein products (Bahari et al. 2020; Dehghani et al. 2018; Joukar et al. 2017; Moghaddas Kia et al. 2018). In a study by Dehghani et al. (2018), the effects of PG coatings loaded with clove and thyme essential oil emulsions on increasing the shelf life of rainbow trout fillets during cold storage were investigated. They concluded that using PG more than doubled the increase in the shelf life (Dehghani et al. 2018).

Probiotics, encompassing bacterial and fungal species such as *L. sakei* , are known to offer health benefits to humans by promoting beneficial microbiota balance. These bioactive metabolites demonstrate versatile functions in both the processing and preservation of various fermented and non‐fermented food products (Pourjafar et al. 2023). *L. sakei* is a facultative anaerobic, Gram‐positive lactic acid bacterium and is one of the most important bacterial species involved in the preservation and fermentation of meat. This bacterium is part of the natural flora of fresh meat and becomes the dominant flora during the anaerobic storage of meat. The essential role of *L. sakei* in meat preservation and fermentation is mainly due to the production of lactic acid and the synthesis of inhibitory compounds against pathogenic and spoilage bacteria (Abdul Hakim et al. 2023). *L. sakei* is classified as a Generally Recognized as Safe (GRAS) microorganism and can be used as a bio‐preservative in meat product packaging (Beristain‐Bauza et al. 2017).

One key indicator for assessing the quality of beef is the microbial load during storage. Additionally, chemical changes in the meat, such as alterations in pH, directly influence its structural, sensory, and physicochemical properties (Bahari et al. 2020). Despite numerous studies on the use of gums in meat preservation, there has been no investigation into the combination of PG with probiotics to date. Therefore, the objective of the present study is to evaluate the effect of PG coatings, at various concentrations, incorporating *L. sakei* bacteria on beef quality. This evaluation includes microbiological, chemical, and organoleptic properties during 8 days of refrigerated storage at 4°C.

## 2 Materials and Methods

### 2.1 Preparation and Formulation of the Bacterial Strain

The probiotic bacterium used in this study was the *L. sakei* species (PTCC 1712), which was obtained in lyophilized form from the Iranian Research Organization for Science and Technology (Bacterial and Fungal Collection). According to the instructions provided by this center for the preparation of the bacterial strain, it was cultured twice on MRS agar medium (Merck, Germany). The resulting culture medium was incubated at 37°C for 48 h. The turbidity method was employed to determine the initial number of bacteria, based on the half‐McFarland standard. The absorbance was measured using a spectrophotometer at a wavelength of 600 nm, and the initial number of bacteria was determined. Then, a quantity equivalent to 5 Log_10_ CFU/g of the residual cells was incorporated into the PG coating solution.

### 2.2 Preparation of the PG Coating Solution Containing *L. sakei*

The PG was purchased from a reputable spice shop in Tehran. The dried gum pieces were completely ground using an electric grinder and then dissolved in water. To separate the gum impurities (dry leaves and husks in the gum), the resulting solution was passed through a cotton cloth. To hydrate all the gum particles, the filtered solution was kept at room temperature for 24 h, and then the solution was placed in a centrifuge at 20°C for 15 min to completely separate the soluble phase. The separated phase was completely dried and powdered using a freeze‐dryer. To prepare a 1% gum solution, 10 g of PG powder was added to 1000 mL of distilled water, and it was homogenized on a magnetic stirrer for 30 min; also a 2% gum solution was prepared by the same method (Amini Rastabi and Mirzaey 2018).

Subsequently, to formulate a PG coating solution enriched with *L. sakei* , 5 mL of a suspension containing 5 Log_10_ CFU/g of the bacterial strain was introduced into the gum solution. The mixture was then subjected to stirring with a heated magnetic stirrer at 25°C for 15 min to ensure uniform dispersion (Ebrahimi et al. 2018).

### 2.3 Preparation of Beef and Coating of the Studied Treatments

Fresh beef loin was purchased 12–24 h after slaughter (completion of the rigor mortis process) from one of the sales units of a slaughterhouse in Tehran and transferred to the laboratory in 30 min, in the presence of ice gel bags. In the laboratory, the purchased meat was aseptically cut into pieces weighing 80–100 g and approximately 3–4 cm in diameter. The studied treatments were divided into five groups for the tests (Mojaddar Langroodi and Tajik 2017): Group 1: Meat pieces that received no treatment and were only immersed in sterile distilled water (control group). Group 2: Meat pieces immersed in 1% PG solution (PG 1%). Group 3: Meat pieces immersed in 2% PG solution (PG 2%). Group 4: Meat pieces immersed in 1% PG solution containing *L. sakei* bacteria (PG1% + *L. sakei* ). Group 5: Meat pieces immersed in 2% PG solution containing *L. sakei* bacteria (PG 2% + *L. sakei* ). The treatment groups were immersed in the coating solution for 1 min. Then, the samples were removed from the solution and placed on a filter for 1 h at room temperature to allow the excess solution to drain and the coating layer to form. After that, the samples were individually packaged in polyethylene bags and stored in a refrigerator (4°C). On days 0, 2, 4, 6, and 8 of the storage periods, three pieces from each treatment were randomly selected and tested for microbiological, chemical, and sensory parameters (Dini et al. 2020).

### 2.4 Microbial Analyses

For the enumeration of microorganisms, 10 g of the beef loin sample was added to 90 mL of peptone buffer, and the resulting suspension was homogenized using a stomacher (stomacher 400, Interscience, France) (Ortuño et al. 2014). For the enumeration of aerobic mesophilic bacteria, the pour plate method was used on Plate Count Agar (Merck, Germany), with the plates incubated at 37°C for 48 h. For the enumeration of psychrotrophic bacteria, the pour plate method was used on Plate Count Agar, and the plates were incubated at 7°C for 10 days. After the incubation period, the colonies were counted (Dini et al. 2020). For the enumeration of Enterobacteriaceae, the samples were inoculated onto Violet Red Bile Glucose Agar (Merck, Germany) using the pour plate method, with the plates incubated at 37°C. After 24 h, the grown colonies were counted (Jones et al. 1997). The enumeration of Lactic Acid Bacteria (LAB) was performed by inoculating the samples onto MRS Agar using the pour plate method, followed by incubation at 30°C for 24–48 h (International Standard, ISO 13721 1995).

### 2.5 Chemical Analyses

#### 2.5.1 Measurement of pH

To determine the pH of the beef loin samples, 10 g of the sample was transferred to a 250 mL beaker containing 90 mL of distilled water and thoroughly homogenized. The pH of the samples was then measured using a digital pH meter (Mettler Toledo, Seven Easy, USA) (Fan et al. 2009).

#### 2.5.2 Measurement of Total Volatile Basic Nitrogen (TVB‐N)

The total volatile basic nitrogen was measured using the Kjeldahl apparatus following the method described by Jeon et al. (2002). For this purpose, 10 g of the homogenized sample, along with 2 g of magnesium oxide and 250 mL of distilled water, were transferred to a Kjeldahl distillation flask. Several glass beads were added to the flask to prevent foaming during boiling. After 30 min of heating and distillation of the nitrogen compounds, the resulting vapors were collected in a 250 mL beaker containing 2% boric acid solution and methyl red indicator. The contents of the beaker were then titrated with 0.1 N sulfuric acid until a stable pink color appeared. The total volatile basic nitrogen was reported as milligrams of nitrogen per 100 g of meat, according to the following formula (Jeon et al. 2002):

TVB−N=Volume of sulfuric acid consumed×14.

#### 2.5.3 Measurement of Thiobarbituric Acid Reactive Substances (TBARS)

For the measurement of thiobarbituric acid, first, 2 g of the sample was analyzed according to the method described by Amaral et al. (2018), using a spectrophotometer (HATCH, USA, model DR6000) at a wavelength of 532 nm.

#### 2.5.4 Measurement of Protein Oxidation Index (Protein Carbonyl) Section

To measure the protein oxidation index, the method described by Mercier et al. (1998) was used. For this purpose, 2 g of the beef loin sample was transferred to a 50 mL falcon tube, to which 20 mL of 0.15 M potassium chloride solution was added. The contents of the falcon tube were then homogenized using a homogenizer at 10,000 rpm for 30 s. From the homogenized solution, two 1 mL samples were taken and transferred to two 15 mL falcon tubes. Then, 1 mL of 20% w/v trichloroacetic acid (TCA) solution was added to each falcon tube, and the contents were mixed using a shaker at 2500 rpm. To separate and precipitate the carbonyl groups and proteins, the tubes were centrifuged at 2000 RCF for 10 min, resulting in a liquid phase at the top and a protein and carbonyl phase at the bottom. The liquid phase of each tube was removed and discarded using a pipette. The tubes were labeled as A and B. Tube A was used for measuring the protein concentration, and tube B was used for measuring the carbonyl concentration. Specifically, 2 mL of 2 N hydrochloric acid was added to tube A, while 2 mL of DNPH solution (2% in 2 N hydrochloric acid) was added to tube B. The tubes were placed in a dark place for 1 h, and then the contents of each tube were homogenized using a homogenizer at 2500 rpm for 15 s. Next, 2 mL of 20% TCA solution was added to the tubes, and after mixing, the tubes were centrifuged at 2000 RCF for 10 min to separate the contents into two phases. The liquid phase of each tube was removed and discarded using a pipette. To wash the resulting precipitates, 4 mL of an ethanol‐ethyl acetate solution (1:1 v/v) was added to each tube, and the contents were homogenized at 2500 rpm for 10 s. The tubes were then centrifuged at 2000 RCF for 10 min, and the liquid phase was removed. This washing step with the ethanol‐ethyl acetate solution, homogenization, and centrifugation was repeated.

To dissolve the final pellet, 4 mL of 6 M guanidine hydrochloride solution (in 20 mM sodium phosphate buffer) was added to each tube, and the tubes were placed in the refrigerator for 12 h. To dissolve any remaining suspended particles, the tubes were incubated in a 50°C water bath for 1 h and then centrifuged at 7000 RCF for 10 min. To measure the protein concentration, the supernatant from tube A was diluted 1:4 with distilled water, and the absorbance was measured using a spectrophotometer at 280 nm. We measure the protein concentration according to the following equation and multiply the resulting number by 5.

$$X\;\left(\mathrm{mg}\;\text{Protein}/\mathrm{mL}\;\text{sample}\right)=\mathrm{ABS}280/0.2265.$$

To measure the carbonyl concentration, the absorbance of the supernatant from tube B was measured using a spectrophotometer at 370 nm. The carbonyl concentration was calculated using the following equation.

$$\begin{array}{c} K\;\left(\text{nmol carbonyl}/1\;\mathrm{cc}\right)=\left({\mathrm{ABS}}_{370}/E.L\right)\times 10^{6}\\ =\left({\mathrm{ABS}}_{370}/21000\right)\times 10^{6}. \end{array}$$

Finally, the amount of carbonyl was calculated as nmol carbonyl per mg of protein using the following formula (Mercier et al. 1998):

$$\text{Carbonyl}\;\left(\text{nmol carbonyl}/\mathrm{mg}\;\text{protein}\right)=K/X.$$

### 2.6 Organoleptic Properties

Four properties—color, odor, texture, and overall acceptability—were considered as the sensory evaluation indices, and the evaluation method was based on the 9‐point scoring system described by Cai et al. (2014). In this method, 10 trained panelists were asked to evaluate the samples for the mentioned attributes and score them between 1 and 9. A score of 9 represented “extremely desirable,” 1 represented “completely unacceptable,” with a score of 5 defined as the acceptance limit (Cai et al. 2014).

### 2.7 Statistical Analysis

All tests were performed in triplicate. Data analysis was carried out using SPSS software version 26 (SPSS Inc., Chicago, IL, USA) and the Repeated Measurement ANOVA method. If a significant difference was found between the means, the Tukey post hoc test was used for pairwise comparisons. For the comparison of sensory properties such as color, odor, texture, and overall acceptability among the test groups, the Kruskal–Wallis test was used, and for pairwise comparisons, the Mann–Whitney *U* Test with Bonferroni Correction was employed. The results were expressed as mean and standard deviation. The statistical significance level was set at *p* < 0.05. GraphPad Prism software version 9 (GraphPad Software, La Jolla, CA, USA) was used for plotting the graphs. The Ethics Committee of Amol University of Special Modern Technologies, Iran, approved this study (IR.AUSMT.REC.1403.06).

## 3 Results

### 3.1 Effect of PG‐Based Coating Enriched by *L. sakei* on the Microbial Properties of Beef

#### 3.1.1 Mesophilic Bacteria Count

**FIGURE 1:**
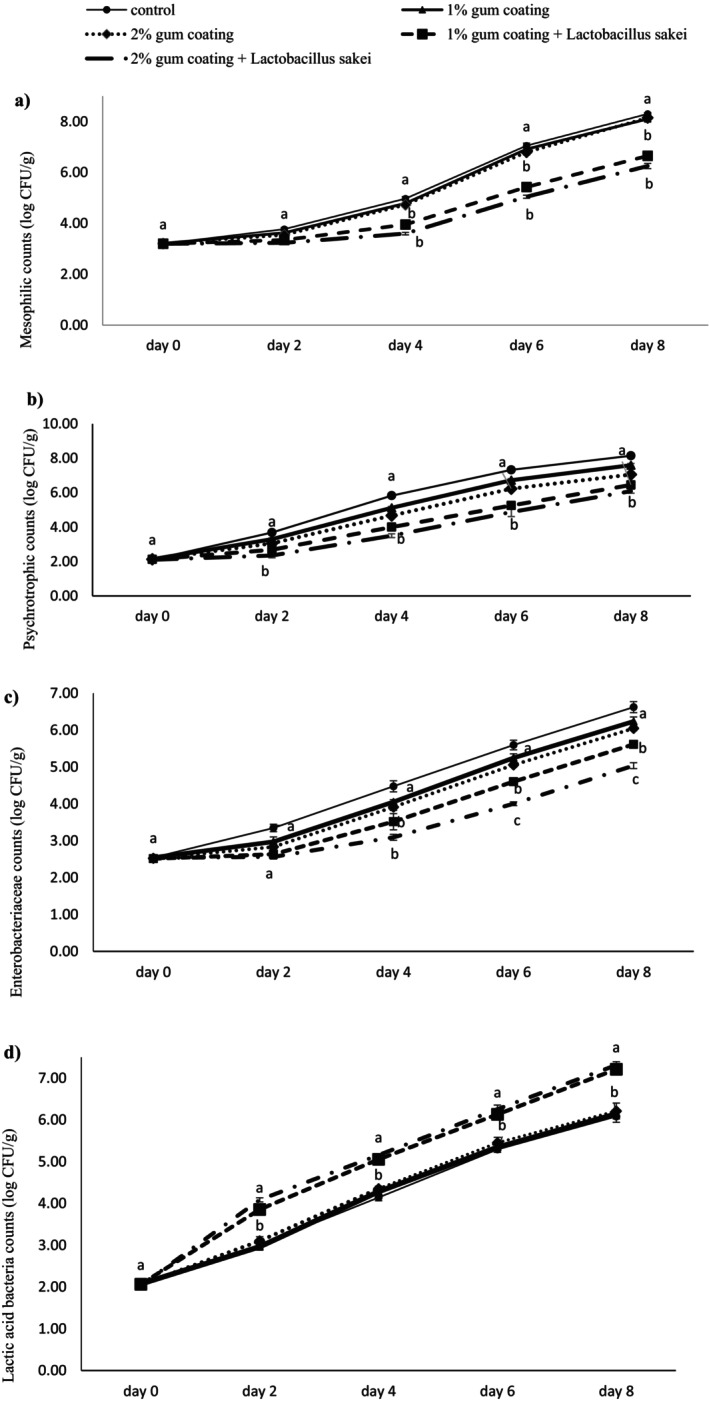
Microbial analysis results in different beef treatments coated with PG and the probiotic bacterium *L. sakei* during 8‐day refrigerated storage. Dissimilar letters on each day reveal significant differences between treatment groups (*p* < 0.05).

The results of the mesophilic bacteria count in the different beef loin treatments during refrigerated storage are presented in Figure 1. The results show that the mean mesophilic count at different time points differed significantly (*p* < 0.05), and the mean mesophilic count increased over time in all groups. The mean mesophilic count on day 0 was at the same level in all groups and did not differ significantly, but it increased over time. On days 2, 4, 6, and 8, the mean mesophilic count in the control, 1% gum coating, and 2% gum coating groups did not differ significantly. Still, these 3 groups revealed a significant difference compared to the “1% gum coating + *L. sakei* ” and “2% gum coating + *L. sakei* ” groups (*p* < 0.05). The treatments in groups 4 and 5 had the greatest effect on controlling the mesophilic microbial population during the 8‐day refrigerated storage of the beef loin. The first three groups reached a mesophilic count of log 7 on day 6 (Christian and Roberts 1986), while the last two groups containing the probiotic *L. sakei* in 1% and 2% PG coatings did not reach log 7 even by day 8 (Figure 1a).

#### 3.1.2 Psychrotrophic Bacteria Count

The changes in the mean psychrotrophic bacteria count over time also showed a significant difference (*p* < 0.05) between the time points, and the mean psychrotrophic count increased over time in all groups. The mean psychrotrophic count on day 0 did not differ significantly among the groups. However, on days 2, 4, 6, and 8, the mean psychrotrophic count in the control, 1% gum coating, and 2% gum coating groups presented a significant difference compared to the “1% gum coating + *L. sakei* ” and “2% gum coating + *L. sakei* ” groups (*p* < 0.05). The overall trend of the mean psychrotrophic count was ascending in all groups over time, but the rate of this increase was lower in the “2% gum coating + *L. sakei* ” group compared to the other groups throughout the storage period (Figure 1b).

#### 3.1.3 Enterobacteriaceae Count

The changes in the mean Enterobacteriaceae count also indicate that the mean Enterobacteriaceae count at different time points differed significantly (*p* < 0.05), and the mean count of these bacteria increased over time in all groups. The mean Enterobacteriaceae count on day 0 did not differ significantly among the groups, but on days 2, 4, 6, and 8, the difference between the control group and the “1% gum coating + *L. sakei* ” and “2% gum coating + *L. sakei* ” groups was significant. The overall trend of the mean Enterobacteriaceae count was growing in all groups over time. On days 4, 6, and 8, the mean Enterobacteriaceae count in the “2% gum coating + *L. sakei* ” group was significantly lower than all other groups (*p* < 0.05) (Figure 1c).

#### 3.1.4 LAB Count

The mean LAB count differed significantly over time (*p* < 0.05). The mean LAB count on day 0 did not differ significantly among the groups, but on days 2, 4, 6, and 8, the difference in LAB count was significant in all groups. The overall trend of the mean LAB count was upward in all groups over time, but the trend of the control, 1% gum coating, and 2% gum coating groups did not differ significantly over time. On days 2, 4, 6, and 8, the mean LAB count in the “1% gum coating + *L. sakei* ” and “2% gum coating + *L. sakei* ” groups was significantly higher than the other three groups (Figure 1d).

### 3.2 Effect of PG‐Based Coating Enriched by *L. sakei* on the Chemical Properties of Beef

#### 3.2.1 pH Value

Table 1 presents the changes in the mean pH over time. The results show that the mean pH increased over time in all groups (*p* < 0.05). The mean pH on day 0 did not differ significantly among the groups, but on days 2, 4, 6, and 8, the pH difference between the groups was significant. The overall trend of the mean pH was ascending in all groups over time, but on days 2, 4, 6, and 8, the mean pH in the “1% gum coating + *L. sakei* ” and “2% gum coating + *L. sakei* ” groups was significantly lower than in the other groups.

**TABLE 1:** Changes in the amount of pH of meat over the time storage at 4°C (mean ± SD).

Group	Day 0	Day 2	Day 4	Day 6	Day 8	*p*
Control	5.71 ± 0.08^Aa^	5.98 ± 0.09^Ab^	6.13 ± 0.09^Ac^	6.34 ± 0.08^Ad^	6.57 ± 0.05^Ae^	0.001
PG 1%	5.70 ± 0.04^Aa^	5.89 ± 0.09^Ab^	6.05 ± 0.07^ABc^	6.20 ± 0.05^Ad^	6.46 ± 0.06^ABe^	0.001
PG 2%	5.71 ± 0.03^Aa^	5.85 ± 0.12^Ab^	6.01 ± 0.14^BCc^	6.14 ± 0.04^Ad^	6.38 ± 0.09^BCe^	0.001
PG 1% + *L. sakei*	5.69 ± 0.18^Aa^	5.80 ± 0.05^ABab^	5.89 ± 0.02^Cb^	5.96 ± 0.07^Ac^	6.09 ± 0.08^Cc^	0.001
PG 2% + *L. sakei*	5.69 ± 0.15^Aa^	5.73 ± 0.13^Ba^	5.82 ± 0.03^Da^	5.90 ± 0.05^Bab^	5.98 ± 0.03^Db^	0.001

*Note:* Dissimilar Capital letters in each column reveal a significant difference among different groups. Dissimilar lowercase letters in each row reveal a significant difference among periods. PG 1% stands for 1% gum coating, PG 2% stands for 2% gum coating, PG 1% + *L. sakei* stands for 1% gum coating + *L. sakei* , and PG 2% + *L. sakei* stands for 2% gum coating + *L. sakei* .

#### 3.2.2 Total Volatile Base Nitrogen (TVB‐N)

Table 2 reports the changes in the mean TVB‐N over time in the groups. The mean TVB‐N increased over time in all groups (*p* < 0.05). The mean TVB‐N did not differ significantly among the groups on day 0, but on days 2, 4, 6, and 8, the TVB‐N difference between the groups was significant. The overall trend of the mean TVB‐N was rising in all groups over time, but the rate of this increase in the “1% gum coating + *L. sakei* ” and “2% gum coating + *L. sakei* ” groups was significantly lower than the other groups. In the control group, the TVB‐N value exceeded 15 (Holman et al. 2021) from day 4, indicating spoilage, while in the 1% gum + *L. sakei* group, it reached this value on day 6, and in the last group (2% gum + probiotic), it reached this value on day 8. Therefore, the last group delayed meat spoilage by 4 days.

**TABLE 2:** Changes in the amount of TVB‐N (mg N/g) of meat over the time storage at 4°C (mean ± SD).

Group	Day 0	Day 2	Day 4	Day 6	Day 8	*p*
Control	8.00 ± 0.50^Aa^	13.35 ± 0.17^Ab^	19.50 ± 1.50^Ac^	25.85 ± 0.79^Ac^	32.33 ± 1.04^Ad^	0.001
PG 1%	8.01 ± 0.07^Aa^	12.78 ± 0.51^ABb^	17.97 ± 0.64^Ac^	23.95 ± 1.30^Ad^	30.48 ± 0.97^Ae^	0.001
PG 2%	8.03 ± 0.06^Aa^	11.33 ± 0.33^ABab^	16.45 ± 0.35^Ab^	22.83 ± 1.04^Ac^	29.02 ± 0.08^Ad^	0.001
PG 1% + *L. sakei*	8.00 ± 0.05^Aa^	9.30 ± 0.28^BCab^	13.38 ± 0.15^Bbc^	17.30 ± 0.28^Ac^	22.20 ± 0.23^Ad^	0.001
PG 2% + *L. sakei*	8.01 ± 0.01^Aa^	8.10 ± 0.17^Ca^	11.10 ± 0.13^Bb^	14.45 ± 0.26^Ac^	18.30 ± 0.28^Ad^	0.001

*Note:* Dissimilar Capital letters in each column reveal a significant difference among different groups. Dissimilar lowercase letters in each row reveal a significant difference among periods. PG 1% stands for 1% gum coating, PG 2% stands for 2% gum coating, PG 1% + *L. sakei* stands for 1% gum coating + *L. sakei* , and PG 2% + *L. sakei* stands for 2% gum coating + *L. sakei* .

#### 3.2.3 Thiobarbituric Acid‐Reactive Substances (TBARS)

Table 3 indicates that the mean TBARS differed significantly over time (*p* < 0.05), and the mean TBARS increased over time in all groups. The mean TBARS on day 0 did not differ significantly among the groups, but on days 2, 4, 6, and 8, the TBARS difference between the groups was significant. The overall trend of the mean TBARS was increasing in all groups over time. On day 8, the mean TBARS in the “1% gum coating + *L. sakei* ” and “2% gum coating + *L. sakei* ” groups was significantly lower than in the other groups, and the upward slope of this mean was generally lower in these two groups compared to the other groups.

**TABLE 3:** Changes in the amount of TBARS (mg MDA/kg) of meat over the time storage at 4°C (mean ± SD).

Group	Day 0	Day 2	Day 4	Day 6	Day 8	*p*
Control	0.32 ± 0.01^Aa^	1.25 ± 0.10^Ab^	2.18 ± 0.15^Ac^	3.23 ± 0.28^Ad^	4.12 ± 0.11^Ae^	0.001
PG 1%	0.32 ± 0.02^Aa^	1.05 ± 0.05^Bb^	1.82 ± 0.15^ABc^	2.83 ± 0.07^Bd^	3.75 ± 0.10^Ae^	0.001
PG 2%	0.32 ± 0.01^Aa^	0.95 ± 0.05^Cb^	1.45 ± 0.20^Bc^	2.45 ± 0.10^Bd^	3.18 ± 0.06^Ae^	0.001
PG 1% + *L. sakei*	0.32 ± 0.02^Aa^	0.68 ± 0.03^Cb^	0.99 ± 0.07^Bc^	1.55 ± 0.26^Bd^	2.55 ± 0.10^Ae^	0.001
PG 2% + *L. sakei*	0.32 ± 0.01^Aa^	0.48 ± 0.15^Ea^	0.70 ± 0.05^Cab^	1.00 ± 0.05^Cc^	2.16 ± 0.14^Bd^	0.001

*Note:* Dissimilar Capital letters in each column reveal a significant difference among different groups. Dissimilar lowercase letters in each row reveal a significant difference among periods. PG 1% stands for 1% gum coating, PG 2% stands for 2% gum coating, PG 1% + *L. sakei* stands for 1% gum coating + *L. sakei* , and PG 2% + *L. sakei* stands for 2% gum coating + *L. sakei* .

#### 3.2.4 Protein Carbonyl

The results in Table 4 show that the mean protein carbonyl differed significantly over time (*p* < 0.05), and the mean protein carbonyl rose over time in all groups. The mean protein carbonyl on day 0 did not differ significantly among the groups, but the difference was significant on days 2, 4, 6, and 8. The overall trend of the mean protein carbonyl was upward in all groups over time. Still, this mean was significantly lower in the “1% gum coating + *L. sakei* ” and “2% gum coating + *L. sakei* ” groups compared to the other groups.

**TABLE 4:** Changes in the amount of protein carbonyl (nmol carbonyl/mg protein) of meat over the time storage at 4°C (mean ± SD).

Group	Day 0	Day 2	Day 4	Day 6	Day 8	*p*
Control	0.71 ± 0.01^Aa^	1.07 ± 0.04^Ab^	1.39 ± 0.07^Ac^	1.81 ± 0.04^Ad^	2.05 ± 0.09^Ae^	0.001
PG 1%	0.72 ± 0.09^Aa^	0.98 ± 0.12^ABa^	1.28 ± 0.03^Ab^	1.65 ± 0.00^Ab^	1.93 ± 0.04^Ab^	0.001
PG 2%	0.71 ± 0.05^Aa^	0.89 ± 0.04^ABa^	1.19 ± 0.03^Ab^	1.55 ± 0.03^Ab^	1.85 ± 0.11^Ab^	0.001
PG 1% + *L. sakei*	0.71 ± 0.05^Aa^	0.80 ± 0.05^Ba^	1.00 ± 0.05^Bb^	1.22 ± 0.11^Bb^	1.52 ± 0.09^Bb^	0.001
PG 2% + *L. sakei*	0.71 ± 0.05^Aa^	0.75 ± 0.05^Ba^	0.87 ± 0.07^Ba^	1.08 ± 0.10^Cb^	1.33 ± 0.08^Cb^	0.001

*Note:* Dissimilar Capital letters in each column reveal a significant difference among different groups. Dissimilar lowercase letters in each row reveal a significant difference among periods. PG 1% stands for 1% gum coating, PG 2% stands for 2% gum coating, PG 1% + *L. sakei* stands for 1% gum coating + *L. sakei* , and PG 2% + *L. sakei* stands for 2% gum coating + *L. sakei* .

### 3.3 Effect of PG‐Based Coating Enriched by *L. sakei* on the Organoleptic Characteristics of Beef

#### 3.3.1 Color Investigation

**FIGURE 2:**
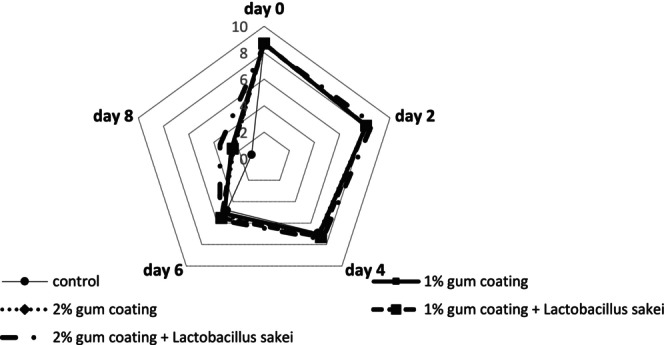
Sensory evaluation results (color) in different beef treatments coated with PG and the probiotic bacterium *L. sakei* during 8‐day refrigerated storage.

Figure 2 demonstrates that the color attribute had a descending trend in all groups. The color decline in the control group was greater than in the other groups, and on day 8, the score of this group reached the lowest value. The rate of color reduction in the 2% gum coating + *L. sakei* group had a gentler slope compared to the other groups, and no significant color reduction was observed in this group until day 4. In the control group, the color became unacceptable from day 6, while the other groups reached a score below 5 (acceptance limit) from day 8.

#### 3.3.2 Odor Investigation

**FIGURE 3:**
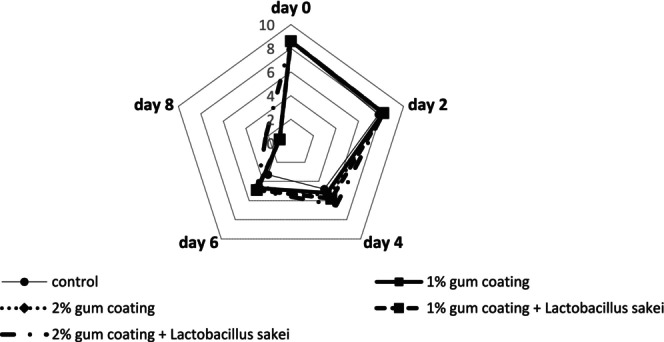
Sensory evaluation results (odor) in different beef treatments coated with PG and the probiotic bacterium *L. sakei* during 8‐day refrigerated storage.

Figure 3 regarding the odor attribute shows that the mean odor score diminished in all groups. The downward trend of this attribute in the control group was greater than in the other groups, and on day 8, it reached the lowest value. However, in the 2% gum coating + *L. sakei* group, the falling trend of the odor score had a gentler slope, and until day 4, the odor score did not drop significantly, but by the end of day 8, it decreased to 2.2. The control group became unacceptable for odor from day 4, but the last group (2% gum + probiotic) reached the acceptance limit of 5 on day 6.

#### 3.3.3 Texture Investigation

**FIGURE 4:**
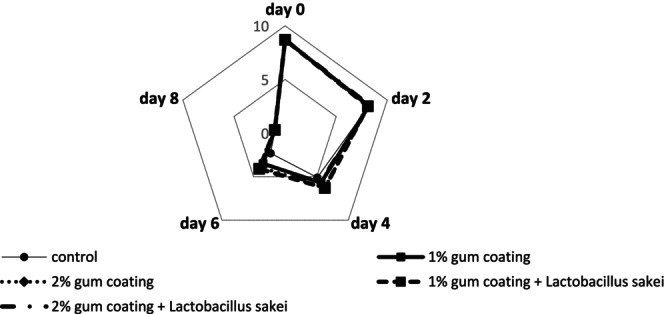
Sensory evaluation results (texture) in different beef treatments coated with PG and the probiotic bacterium *L. sakei* during 8‐day refrigerated storage.

The results of the changes in the texture attribute are displayed in Figure 4. With the increase in storage time, the texture attribute had a declining trend in all groups. The decreasing trend of the texture score in the control group was greater than in the other groups. On day 8, all groups reached the lowest texture score. The 2% gum coating and 1% gum coating + *L. sakei* groups had a similar downward trend in the texture score, and it seems that the slope of the texture score reduction in these two groups was less than in the other groups. The control group became unacceptable for texture from day 4, but the last group (2% gum + probiotic) reached the acceptance limit of 5 on day 6.

#### 3.3.4 Overall Acceptability Investigation

**FIGURE 5:**
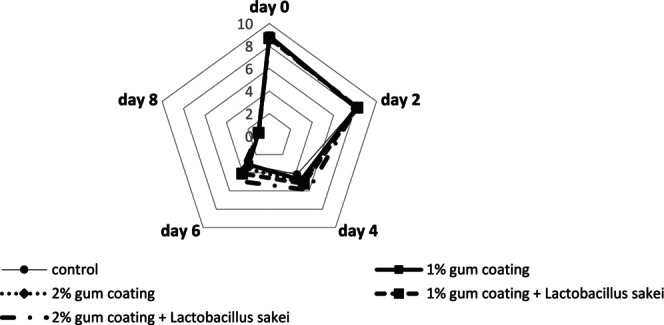
Sensory evaluation results (overall acceptability) in different beef treatments coated with PG and the probiotic bacterium *L. sakei* during 8‐day refrigerated storage.

Figure 5 reveals the mean score of the overall acceptability attribute. The mean overall acceptability on days 0, 2, and 8 did not differ significantly among the groups, and on days 4 and 6, the greatest and least reduction in the overall acceptability attribute was related to the control treatment and the 2% gum coating + *L. sakei* treatment, respectively. The descending trend of the overall acceptability score reached the lowest level in all groups on day 8. The control group became unacceptable for overall acceptability from day 4, but the last group (2% gum + probiotic) reached the acceptance limit of 5 on day 6.

## 4 Discussion

In new food preservation methods, natural preservatives such as essential oils, and organic and plant extracts are preferred over harmful chemical preservatives (Teshome et al. 2022). The presence of protein compounds and high moisture content in meat provides a suitable environment for the growth of spoilage bacteria during storage. Therefore, the level of these microorganisms can be considered as one of the spoilage indicators, and by counting them, the quality of the product can be evaluated during the storage period (Esmaeili et al. 2020).

According to the results of this study, the initial count of mesophilic bacteria was 3.20 log_10_ CFU/g in all treatment groups, indicating the appropriate quality of the beef loin on the first day. However, these bacteria increased in all samples during the storage period. This increase was more intense in the control samples, and all the coated samples, especially those coated with PG and *L. sakei* bacteria, had a lower bacterial count. It was also found in this study that the number of mesophiles reached above the permissible limit (10^7^ CFU/g) by the end of the sixth day in the control, 1% gum, and 2% gum groups, indicating that the shelf life of uncoated meat at refrigerator temperature is less than 6 days. However, using 1% and 2% gum coatings with *L. sakei* bacteria, the average mesophilic count was less than the permissible limit at the end of the eighth day. The observed differences can be attributed to variations in microbial diversity influenced by the presence or absence of PG coatings. Due to the anionic nature of PG, it is unlikely to possess intrinsic antimicrobial properties. Instead, any antimicrobial effects associated with the treatment may be attributed to the presence of *L. sakei* . Furthermore, the coating may function as an effective oxygen barrier, thereby inhibiting the growth of aerobic bacteria (Dehghani et al. 2018).

This result is consistent with the findings of Saffari Samani et al. (2023), who investigated the effect of PG‐based edible coating containing Zataria multiflora Bois's essential oil on the quality enhancement and shelf‐life improvement of fresh buffalo meat (Saffari Samani et al. 2023).

An important part of the microbial flora of meat stored in the refrigerator belongs to the proliferation of Gram‐negative psychrotrophic bacteria (Dehghani et al. 2018). According to the observations in this study, the number of these bacteria was at a similar level on day 0 in the different treatments and within the range from 2.10 to 2.13 log_10_ CFU/g. The investigations revealed that the number of psychrophiles had an ascending trend during the storage period in all treatments. However, the intensity of this trend was different in various treatment groups, so the highest upward trend was related to the control group, and the slowest trend was related to the 2% PG coating group containing the probiotic *L. sakei* . In addition to the bacteriocin‐producing property of *L. sakei* (Leroy and De Vuyst 2001), which can affect the growth of other microorganisms, considering that Pseudomonas are highly aerobic bacteria and their growth, as well as survival, is problematic in the absence of oxygen, it can be inferred that the 1% and 2% gum coatings were also among the influential factors on their growth. This result is also in line with the research by Joukar et al. (2017) on the effect of PG coating along with a nano‐emulsion of cinnamon and Shirazi thyme oils on the shelf life of rainbow trout fillets at refrigerator temperature (Joukar et al. 2017).

Considering that the natural origin of many bacteria of the Enterobacteriaceae family is the digestive system of animals, the presence of these bacteria in raw meat and its products can be considered an important sanitary indicator in these products (Mladenović et al. 2021).

The results of this study show that the increasing trend of the count of Enterobacteriaceae in all coated samples compared to the control group was less intense. Also, the 1% and 2% PG coatings containing the probiotic bacterium *L. sakei* had the greatest limiting effect on the growth of Enterobacteriaceae bacteria. The study conducted by Raeisi et al. (2016) revealed that the values of the tested microbial indices increased across all samples during the storage period. However, the use of antimicrobial agents in combination demonstrated a more pronounced effect in preserving the microbial quality of chicken meat samples. Notably, the strongest preservation effect was observed in samples coated with an alginate solution enriched with cinnamon and rosemary essential oils (Raeisi et al. 2016).

A significant factor contributing to meat spoilage is the presence of facultative anaerobic LAB. These microorganisms play a prominent role within the microbial ecosystem of poultry meat, where they constitute a substantial proportion of the overall microbial flora. Their metabolic activity under various storage conditions can accelerate spoilage processes, highlighting their importance in the context of meat preservation challenges. The initial counts of this bacteria were within the range of 2.04–2.07 log_10_ CFU/g, reaching 6.08 log_10_ CFU/g at the end of storage. The investigations indicated that the number of LABs showed an upward trend during the storage period in all treatments. But unlike other bacteria under study, such as mesophiles, Enterobacteriaceae, or psychrotrophic, the slowest upward trend was related to the control, 1% gum, and 2% gum groups, and the highest increasing trend was related to the 2% and 1% PG coating groups containing the probiotic *L. sakei* . The observed trend in the population dynamics of LAB across the various treatments appears to be largely influenced by incorporating the probiotic *L. sakei* into the coatings. This addition, in combination with the endogenous LAB naturally present within the meat's microbial flora, likely serves as a key factor driving these changes.

De Lacey et al. (2014) investigated the impact of agar‐based films enriched with green tea extract and the probiotics *Lactobacillus paracasei* and *Bifidobacterium lactis* on the shelf life of fish fillets. Their findings demonstrated a significant increase in the levels of probiotics and LAB in the coated groups compared to the control treatments, aligning with the results of this study (De Lacey et al. 2014).

The results of this study demonstrated that the pH changes observed during the storage of the treatments followed an ascending trend. This can be attributed to microbial and enzymatic activity, the degradation of protein compounds, and the subsequent production of nitrogenous compounds. In this study, the pH value in all treatments on day 0 was in the range of 5.69–5.71, and this value had an upward trend until the end of the eighth day so that the highest pH value on day 8 was observed in the control, 1% gum, and 2% gum groups. The results showed that the 2% PG coating containing *L. sakei* , followed by the 1% PG coating with the same probiotic, had the most significant effect in mitigating the increasing trend of pH. This effect can be attributed to lactic acid production by *L. sakei* in treatments 4 and 5. This probiotic, by producing bacteriocins and inhibiting the growth of other bacteria, prevents the activity and formation of metabolites that contribute to the elevation of pH, such as amines and volatile compounds. Additionally, the increase in pH during the storage period of the product may be attributed to the activity of meat protease enzymes, which, through the breakdown and degradation of protein compounds, lead to the production of nitrogenous compounds, ultimately resulting in a rise in pH (Pabast et al. 2018).

The results of the study by Fan et al. (2009) on the effect of chitosan coating on the shelf life of carp fish are in line with the present study regarding the increase in pH during the storage period (Fan et al. 2009).

One of the important quality indicators of meat is the TVB‐N index. As the storage period is extended, the rate of protein degradation and breakdown intensifies, resulting in a more extensive occurrence of spoilage (Esmaeili et al. 2020). According to the results of this research, the TVB‐N value in all samples had an upward trend during the meat storage period. However, this trend was more intense in the control, 1% gum, and 2% gum groups, and the coated groups containing the *L. sakei* bacterium experienced a slower trend in the increment of TVB‐N.

The increase in TVB‐N values can be attributed to microbial proliferation and the activity of endogenous enzymes, which lead to protein breakdown and the production of volatile nitrogen compounds. A positive correlation may exist between the rise in TVB‐N levels and the growth of the bacterial population across different samples. Protein degradation by bacteria occurs more rapidly under aerobic conditions, while using edible coatings can create anaerobic environments in the treated samples, thereby inhibiting the rise in TVB‐N values in the coated groups.

The findings of Ranjbar and Azizi (2017) regarding the impact of a gelatin‐carboxymethyl cellulose coating containing mastic essential oil on the microbial, chemical, and sensory properties of chicken fillets showed an increase in TVB‐N values during the storage period, which is consistent with the results observed in the current study (Ranjbar and Azizi 2017).

The oxidation of unsaturated fatty acids produces unstable compounds called hydroperoxides. Hydroperoxides decompose over time and during food storage, creating new compounds such as malondialdehyde. By measuring the amount of malondialdehyde, the TBARS index can be determined (Dehghani et al. 2018). In this study, the TBARS value in all treatments on day 0 was 0.32 mg MDA/kg, and the five treatments did not have significant differences from each other on this day. From the second day through to the eighth day, the TBARS values exhibited an increasing trend in all groups, with the highest TBARS value on the eighth day observed in the control group samples. On the fourth day, the TBARS value in the control group and the 1% and 2% gum coatings showed numbers higher than 1. According to international food standards, the maximum allowable limit of TBARS in meat is 1 mg MDA/kg (Zhang et al. 2019). Based on this criterion, only the samples from group 5 met the TBARS standard by the end of the sixth day. The investigations revealed that, except for day 0, the TBARS value in the control group was significantly different from those in the other samples on all the studied days. The findings of Hakim et al. (2018) on the effect of chitosan coatings containing pennyroyal essential oil on the shelf life of chicken fillets indicated that the TBARS index in the coated groups was lower compared to the control group samples (Hakim et al. 2018).

Carbonylation of amino acids is a completely harmful process as it produces reactive compounds and leads to the degradation of other proteins. The measurement of free carbonyl groups formed through the oxidation of amino acids is a key indicator for assessing protein oxidation (Guyon et al. 2016). According to the results of this study, the average protein carbonyl content in all samples on the initial day was approximately at the same level (0.71 nmol/mg protein), and they did not have a significant difference. From the fourth day until the end of the storage period, the changes in this index had an upward trend in all treatments. However, these changes exhibited a more pronounced increase in the control group samples, while the coated groups showed a slower rate of protein carbonyl accumulation. In addition to the antimicrobial properties of *L. sakei* , which include the production of bacteriocins and antimicrobial compounds, it can be inferred that the protective effects of the 1% and 2% PG coatings have delayed the protein oxidation process.

The results indicated that initially and on day 0, all samples from the five treatments received high scores for sensory characteristics. This is entirely expected, given the freshness of the samples at the start of the study.

Gradually, throughout the storage period, the sensory scores of the samples showed a declining trend. By the fourth day, the scores had nearly reached the acceptance threshold, and by the eighth day, none of the samples from the five treatments received an acceptable sensory score in terms of color, odor, texture, or overall acceptability. The reduction in sensory scores during the meat storage period is a natural phenomenon, which can be attributed to factors such as increased bacterial activity, oxidation reactions, dehydration, and enzymatic and intracellular processes occurring during storage.

The results of Saffari Samani et al. (2023) in their study on the effect of PG edible coating containing Shirazi thyme essence on enhancing the quality and extending the shelf life of fresh buffalo meat demonstrated that, in terms of sensory evaluation, the coated treatments received higher scores compared to the control treatment (Saffari Samani et al. 2023).

Vital et al. (2016), in the study examining the effect of alginate‐based edible coating containing rosemary essential oil on the characteristics of beef, reported that the trend of water loss and color loss in the coated treatments compared to the control group was significantly less severe, and the coatings had a significant effect on the consumer's perception of odor, taste, and overall acceptance of beef, which is consistent with our results (Vital et al. 2016). Also, the results of Joukar et al. (2017) in the study exploring the effect of an antimicrobial coating based on PG on the shelf life of rainbow trout in the refrigerator were consistent with the results of this study (Joukar et al. 2017).

## 5 Conclusion

The findings of this research highlight the potential benefits of utilizing natural edible coatings, such as Persian gum, in the packaging of fresh meat. One notable advantage is their ability to partially limit the exchange of respiratory gases, contributing to preserving the meat's quality and freshness during storage. In addition to their gas‐barrier properties, these coatings exhibit remarkable carrier capacity, enabling the incorporation of antimicrobial biological agents. This functionality provides a novel approach to integrating bio‐preservatives directly into the packaging material. Specifically, the study demonstrates that coatings enriched with the probiotic bacterium *L. sakei* can enhance the antimicrobial properties of the packaging. This innovation leverages the probiotic's natural preservative effects, further extending the shelf life and ensuring the safety of fresh meat products. The findings of this study indicate that the application of natural edible coatings containing probiotics can significantly delay the spoilage process during the storage period. Specifically, the study demonstrated that a 2% PG coating containing the probiotic *L. sakei* extended the shelf life of coated beef compared to the control group. The beef samples demonstrated the longest and shortest shelf life both chemically and microbiologically in the PG 2% + *L. sakei* group (over 8 days) and the control group (less than 4 days for chemical properties and less than 6 days for microbial properties), respectively. Therefore, the recommended refrigerated shelf life is a maximum of 4 days for uncoated beef and 8 days for coated beef. Furthermore, among the tested coatings, the 2% PG coating containing *L. sakei* had the most pronounced effect on the studied indices, followed by the 1% PG coating containing the bacterium, the 2% PG coating, and finally the 1% PG coating. The application of these coatings, particularly the 2% PG coating with *L. sakei* , presents a promising approach to producing safe, high‐quality meat products without the use of chemical preservatives. This method ensures enhanced preservation while delivering probiotic benefits to consumers, aligning with the growing demand for natural and functional food products. Based on the outcomes of this research, future studies are recommended to explore strategies for increasing the viability of probiotics within the edible coatings. Enhancing probiotic stability could lead to packaging solutions that not only extend shelf life further but also offer stronger probiotic properties, creating a more robust and sustainable food preservation system.

## Author Contributions

**Mohammad Hasan Nemati:** data curation (equal), formal analysis (equal), funding acquisition (lead), investigation (equal), project administration (equal), resources (lead), validation (equal), visualization (equal), writing – original draft (equal), writing – review and editing (equal). **Shohreh Alian Samakkhah:** conceptualization (lead), data curation (equal), formal analysis (lead), methodology (lead), project administration (equal), software (lead), supervision (lead), validation (equal), visualization (equal), writing – review and editing (equal). **Razieh Partovi:** conceptualization (equal), data curation (equal), investigation (equal), project administration (equal), resources (equal), writing – original draft (equal), writing – review and editing (equal). **Abbas Isvand:** data curation (equal), methodology (equal), project administration (equal), resources (equal), writing – original draft (equal), writing – review and editing (equal).

## Ethics Statement

The Ethics Committee of Amol University of Special Modern Technologies, Iran, approved this study (IR.AUSMT.REC.1403.06).

## Consent

The authors have nothing to report.

## Conflicts of Interest

The authors declare no conflicts of interest.

## Data Availability

The data underlying the findings of this study can be obtained from the corresponding author upon a reasonable request.
